# Dynamic pulmonary CT perfusion using first-pass analysis technique with only two volume scans: Validation in a swine model

**DOI:** 10.1371/journal.pone.0228110

**Published:** 2020-02-12

**Authors:** Yixiao Zhao, Logan Hubbard, Shant Malkasian, Pablo Abbona, Sabee Molloi

**Affiliations:** Department of Radiological Sciences, University of California Irvine, Irvine, California, United States of America; University of Iowa, UNITED STATES

## Abstract

**Purpose:**

To evaluate the accuracy of a low-dose first-pass analysis (FPA) CT pulmonary perfusion technique in comparison to fluorescent microsphere measurement as the reference standard.

**Method:**

The first-pass analysis CT perfusion technique was validated in six swine (41.7 ± 10.2 kg) for a total of 39 successful perfusion measurements. Different perfusion conditions were generated in each animal using serial balloon occlusions in the pulmonary artery. For each occlusion, over 20 contrast-enhanced CT images were acquired within one breath (320 x 0.5mm collimation, 100kVp, 200mA or 400mA, 350ms gantry rotation time). All volume scans were used for maximum slope model (MSM) perfusion measurement, but only two volume scans were used for the FPA measurement. Both MSM and FPA perfusion measurements were then compared to the reference fluorescent microsphere measurements.

**Results:**

The mean lung perfusion of MSM, FPA, and microsphere measurements were 6.21 ± 3.08 (p = 0.008), 6.59 ± 3.41 (p = 0.44) and 6.68 ± 3.89 ml/min/g, respectively. The MSM (P_MSM_) and FPA (P_FPA_) perfusion measurements were related to the corresponding reference microsphere measurement (P_MIC_) by P_MSM_ = 0.51P_MIC_ + 2.78 (r = 0.64) and P_FPA_ = 0.79P_MIC_ + 1.32 (r = 0.90). The root-mean-square-error for the MSM and FPA techniques were 3.09 and 1.72 ml/min/g, respectively. The root-mean-square-deviation for the MSM and FPA techniques were 2.38 and 1.50 ml/min/g, respectively. The CT dose index for MSM and FPA techniques were 138.7 and 8.4mGy, respectively.

**Conclusions:**

The first-pass analysis technique can accurately measure regional pulmonary perfusion and has the potential to reduce the radiation dose associated with dynamic CT perfusion for assessment of pulmonary disease.

## Introduction

The advent of quantitative computed tomography (CT) imaging techniques has enabled better evaluation of pulmonary disease. Multidetector CT has previously been used for detection of emphysema [1, 2] and the development of the dual-energy CT has improved the differentiation between different contrast materials (i.e. iodine or xenon) and lung parenchyma for assessment of regional perfusion or ventilation [3, 4]. With the capability of imaging regional pulmonary blood volume, CT has been clinically implemented, thus contributing to assessment for pulmonary embolism, chronic obstructive pulmonary disease [5, 6] and lung cancer [7–9].

Despite positive correlation with microsphere perfusion measurement, existing dynamic CT perfusion techniques have been hampered by a number of limitations [10–14]. First, CT perfusion techniques such as the maximum slope model (MSM), derive pulmonary blood flow by monitoring the contrast enhancement over time using several small volumes-of-interest (VOIs) [13]. However, this approach can be limited by short contrast transit time (<1 sec) through the small VOIs, which are inherently subject to contrast material loss from the small compartments over the measurement period. Second, curve-fitting is performed on these time-series data, which requires a large number of consecutive acquisitions for pulmonary blood flow measurement leading to high radiation dose and motion misregistration artifacts [7–9, 13, 15–17]. Thus, the limitations of the existing perfusion models have hampered the widespread use of dynamic CT for assessment of pulmonary disease.

The purpose of this study was to validate a new low-dose dynamic CT perfusion technique based on first-pass analysis (FPA) for perfusion measurement in a swine model, with the MSM technique also implemented for comparison. The FPA CT perfusion technique has previously been validated for myocardial perfusion measurement in phantom and animal studies [18–20] and addressed the underestimation of the flow measurement by imaging the whole heart within each cardiac cycle. The use of the whole organ as the perfusion compartment allows adequate time for image acquisition and contrast accumulation before contrast exits the perfusion compartment[21, 22]. By increasing the perfusion compartment within the spatial domain, it is possible to reduce the temporal sampling to a minimum of only two time points for FPA measurement. The central hypothesis is that pulmonary perfusion can be accurately measured with the CT FPA technique using only two volume scans, as compared to fluorescent microsphere perfusion method as the reference standard [23].

## Material and methods

### Ethics statement

The experimental protocol was approved by the Institutional Animal Care and Use Committee (IACUC, Protocol Number: AUP-18-191) at University of California, Irvine. All studies were performed under isoflurane anesthesia, and all efforts were made to minimize suffering.

### General methods

Each swine was housed in a single cage in the vivarium and food was withdrawn but water was provided for 12 hours before the study. A total of six swine (41.7 ± 10.2 kg, male, Yorkshire) were used for validation of the FPA technique with fluorescent microsphere perfusion measurements. A standard MSM dynamic CT perfusion technique was also used for comparison. All experimental data were successfully acquired between June 2016 and July 2017 and analyzed between September 2016 and December 2018. All authors conducted the experiments and data acquisition. Three authors (Y.Z., L.H., S. Malkasian.) with more than 3 years of medical imaging research experience conducted the data analysis. One author (P.A.) with 19 years of clinical radiology experience, helped with the intubation, anesthesia, catheters placement and other interventional procedures.

### First-pass analysis (FPA) technique

**Fig 1:**
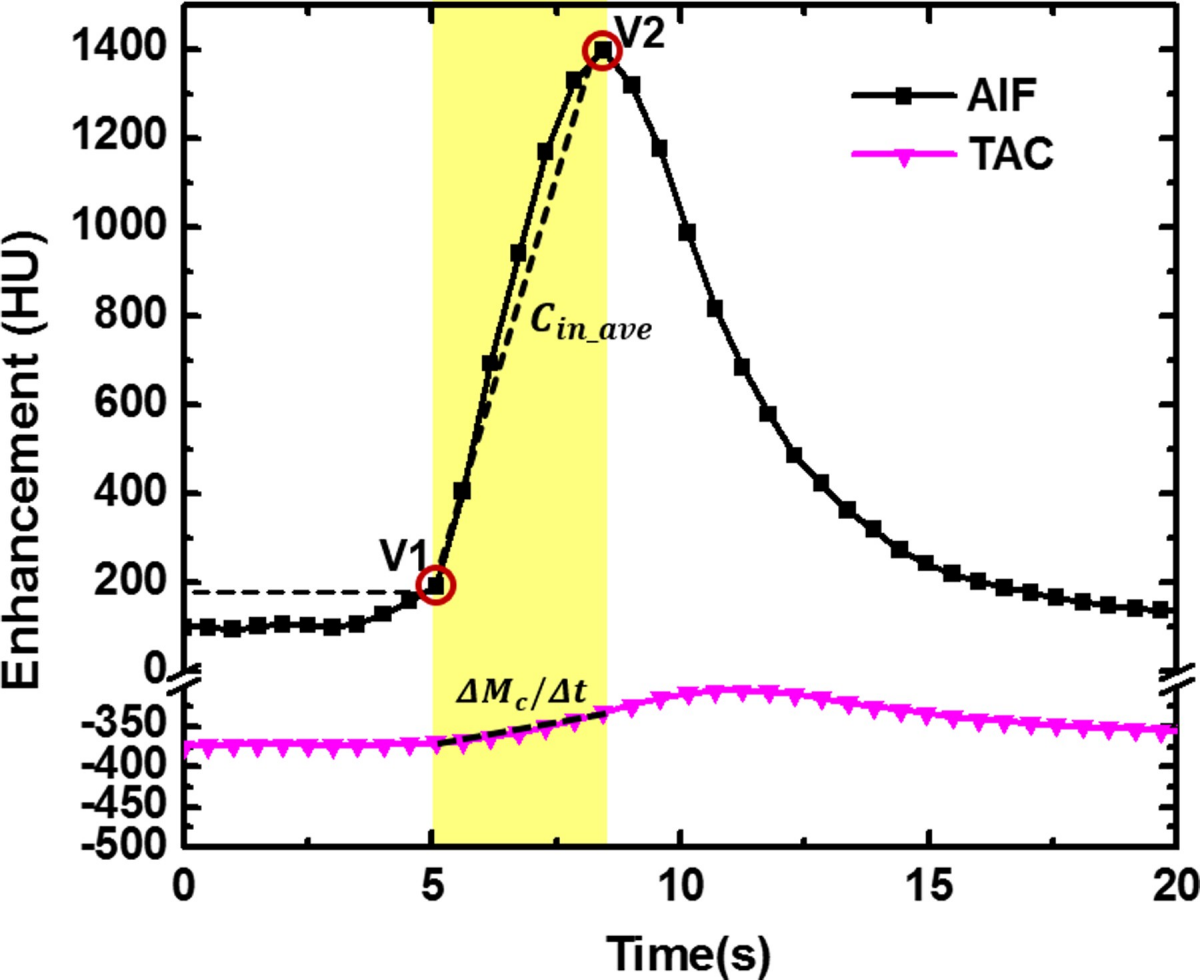
The first-pass analysis acquisition protocol. The lung tissue compartment time attenuation curve (**TAC**) was used to calculate the change in the integrated contrast mass (***ΔM***_***c***_***/Δt***) between the first (**V1**) and second (**V2**) volume scans. The pulmonary arterial input function (**AIF**) was used to estimate the average input concentration (***C***_***in_ave***_) between V1 and V2. The first volume scan (**V1**) was acquired with a threshold of 80 HU above the background blood pool enhancement.

In this study, 20 dynamic CT volume scans were acquired for the MSM technique, but only two volume scans were systematically chosen for the FPA perfusion technique. Specifically, the first volume acquisition (V1) was chosen as the contrast started to enter the compartment with a threshold of 80 HU above the background blood pool enhancement, and the second volume scan (V2) was chosen near the peak of the pulmonary arterial input function as shown in **Fig 1**. The entire lung or lobe was used as the first-pass compartment in the pulmonary perfusion measurement. Assuming no contrast material outflow from the measurement compartment over the measurement period, the lung tissue compartment time attenuation curve (TAC) was used to calculate the change in the integrated contrast mass (***ΔM***_***c***_***/Δt***) between the first (V1) and second (V2) volume scans for the blood flow (***Q***_***ave***_, ***ml/min***) measurement, as shown in ***Eq 1***. The average incoming contrast material concentration ***C***_***in_ave***_, ***mg/ml*** in the pulmonary artery was estimated using the two volume scans as the arterial input function (AIF) [20].

$$Q_{ave}=\frac{1}{C_{in\_ave}}\;\frac{\Delta M_{c}}{\Delta t}$$ (1)

The details of voxel-by-voxel pulmonary perfusion measurement are shown in the **S1 File**.

### Animal preparation

Each swine was premedicated via intramuscular injection of Telazol (4.4mg/kg), Ketamine (2.2 mg/kg), and Xylazine (2.2 mg/kg), intubated (Mallinckrodt, tube 6.0/6.5/7.0, Covidien, Mansfield, MA), then ventilated (Surgivet, Norwell, MA, and Highland Medical Equipment, Temecula, CA) with 1.5–2.5% Isoflurane (Baxter, Deerfield, IL) to maintain anesthesia. Under ultrasound guidance (9L Transducer, Vivid E9, GE 145 Healthcare), four introducer sheaths (AVANTIR, Cordis Corporation, Miami Lakes, FL) were placed in the left jugular vein (intravenous contrast injection), left femoral vein (intravenous fluid and drug administration), right femoral vein (intravenous microsphere injection), and the right femoral artery (mean arterial pressure monitoring), respectively. Two more introducer sheaths were placed in the right jugular vein for the insertion of a pulmonary arterial Swan-Ganz-balloon catheter and for reference microsphere blood withdrawal catheters. Specifically, under fluoroscopic guidance, the Swan-Ganz catheter was placed distally in the left-caudal lobe pulmonary artery branch for induction of balloon occlusion. An additional pulmonary arterial catheter was placed proximally in the left main pulmonary arterial trunk for blood sample withdrawal for microsphere perfusion measurement. Several perfusion conditions were then induced by inflating a balloon in several different locations in the left caudal lobe. ECG, mean arterial pressure (mmHg), end-tidal CO_2_ (mmHg), and O_2_ saturation (%) were monitored and recorded. After the final acquisition, each animal was euthanized with 10 cc of saturated KCL under deep anesthesia and both lungs were resected without draining the blood for tissue sample extraction.

### Reference fluorescent microsphere perfusion measurement

**Fig 2:**
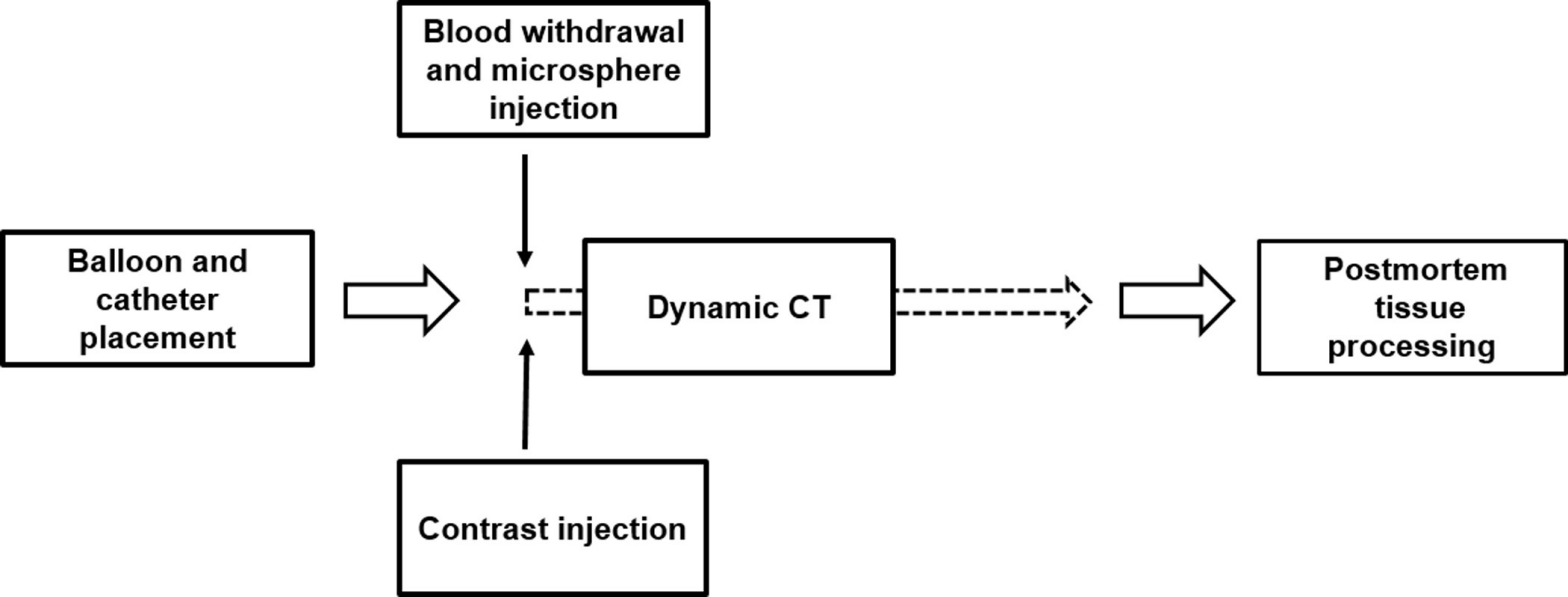
Data acquisition protocol timeline. The protocol includes vascular access using fluoroscopy, dynamic CT acquisition, microsphere measurement, and postmortem tissue processing. The contrast and microsphere injections were made before the continuous dynamic CT scan. The blood sample withdrawal was started 5 seconds prior to the microsphere injection and lasted for 2 minutes.

**Fig 2** shows a summary of data acquisition protocol. The protocol includes vascular access using fluoroscopy, dynamic CT acquisition, microsphere measurement, and postmortem tissue processing. Immediately before each CT perfusion acquisition, one color of fluorescent-labeled microspheres (NuFLOW™, IMT Laboratories, Lawrence, KS) was diluted and injected into the vena cava. A reference blood sample was then withdrawn using a syringe pump at 10ml/min via a pulmonary arterial catheter proximal to the balloon occlusion (GenieTouch; Kent Scientific, Torrington, Conn). The pump was turned on 5 seconds prior to the microsphere injection and was run for a total of 2 minutes. A total of 5 to 8 different color microspheres were used for each animal. Each color of microsphere was used for an independent CT perfusion acquisition. After all acquisitions were completed, the animal was euthanized and 6 to 8 tissue samples (approximately 10g per sample) were excised from different lobes of both lungs. Microsphere (15.5±1.10μm in diameter) analyses were conducted independently by IMT Laboratories [23].

### CT scanning protocol

Each animal was first positioned supine, head first through the gantry (320-slice Aquilion One, Canon Medical Systems, Tustin, CA), and a localization scan was performed with a 16-cm z-coverage to include the balloon placed in the base of the lung. Balloon occlusion was then induced followed by contrast (0.5 or 1 ml/kg, Isovue 370, Bracco Diagnostics, Princeton, NJ) and saline chaser (0.25 or 0.5 ml/kg) injection at 5 ml/s (Empower CTA, Acist Medical Systems, Eden Prairie, MN). For each occlusion, the ventilator was turned off to simulate a breath hold, and ECG-gated volume acquisitions were acquired at functional residual capacity for approximately 20 seconds (100kVp, 200mA or 400mA, 0.35s rotation time, 320 x 0.5 mm collimation, FOV: 240-400mm). Detailed injection and scan parameters are summarized in **Table 1**. Dynamic CT perfusion acquisition was repeated approximately every 20 minutes. All volume acquisitions were reconstructed using 360 degree projection data using a soft tissue kernel (FC07) with a 0.5-mm slice thickness and an adaptive iterative dose reduction 3D (AIDR 3D) reconstruction algorithm. The CT dose index $\left(CTDI_{vol}^{32}\right)$ was recorded and the size-specific dose estimate (SSDE) was also calculated to account for the small effective chest diameter of the swine used in the study [24, 25]. In combination with the chest conversion factor, the effective dose for perfusion measurement with the FPA technique was also estimated [26].

**Table 1:** Acquisition protocols and dose metrics for the FPA and MSM perfusion measurement.

Number of Samples (N)	Contrast (ml/kg)	FPA Perfusion (mGy)		MSM Perfusion (mGy)	
		$CTDI_{vol}^{32}$	SSDE	$CTDI_{vol}^{32}$	SSDE
**200mA (N = 2)**	1	8.2	17.4	138.7	285.8
**400mA (N = 4)**	0.5	26.3	54.2	434.1	894.3

Note.— $CTDI_{vol}^{32}$ is the CT dose index for a 32 cm diameter body dosimetry phantom, SSDE is the size-specific dose estimate based on the effective diameter of the swine in the study (conversion factor: 2.06). Noted that the listed CT dose index for the FPA technique with only 2 volume scans.

### FPA perfusion measurement

Dynamic volume acquisitions were first registered to a single coordinate system by applying a GPU-based affine and deformable registration algorithm [27]. The volume-of-interest was then placed in the pulmonary artery to derive the pulmonary arterial input function **(AIF, Fig 1**). For pulmonary flow measurement, only two volume scans (V1 and V2) were *systematically and retrospectively* selected from the AIF, where V1 was defined as the first volume scan at 80 HU above the baseline blood pool enhancement as the indication of contrast arrival time, and V2 was defined as the peak of the AIF (**Fig 1**). Lung segmentation (lung parenchyma in the FOV) was conducted based on V1. The contrast mass change (***ΔM***_***c***_) in the lung parenchyma and the average arterial input concentration (***C***_***in_ave***_) were used to derive the pulmonary blood flow (***A*.*1–A*.*3***). Finally, to calculate perfusion, the average blood flow was normalized to each voxel (***A*.*4***) and the non-air tissue mass for each voxel was calculated based on V1 (***A*.*5–A*.*6***). The final pulmonary perfusion per voxel was calculated as the voxel flow normalized by voxel mass (***A*.*7***).

In order to derive regional pulmonary perfusion measurements for the validation with microsphere measurements, digital segmentations (ViTAL Images; Canon Medical Systems) were made by a chest radiologist (P.A.) with 19 years of clinical experience, to correspond with the excised tissue samples used for the reference microsphere perfusion measurements. Anatomical landmarks were used as a guide for correspondence between excised tissue samples and digital segmentation. Blood vessels were excluded by excluding voxels greater than 100 HU [1, 28]. The average CT perfusion within each digital segment was compared to the corresponding reference fluorescent microsphere perfusion measurement.

### MSM perfusion measurement

Maximum slope model (MSM) perfusion data were generated using a standard commercial software (ViTAL Images, CT Body Perfusion 4D, Dual-Input Lung Workflow). After automatic registration, small regions-of-interest (ROIs) were placed within the pulmonary trunk and lung parenchyma to generate arterial input functions and tissue attenuation curve, respectively. A parametric pulmonary perfusion map (ml/min/100ml) was then generated. The perfusion maps were rescaled into ml/min/g by using the same mass distribution map (***M***_***x*, *y*, *z***_**, *g/ml***, ***A*.*6***) as the FPA perfusion measurement. Regional perfusion measurements were also averaged using the same regional segmentations as the FPA measurement.

### Statistical analyses

For all microsphere perfusion measurements (including all tissue samples under all perfusion conditions), the combined variance within each subject was compared with the combined variance of measurements between subjects with intra-cluster correlation. To evaluate the performance of the FPA and MSM techniques, linear regression (Pearson’s r) and Bland-Altman analyses were performed as compared to the reference fluorescent microsphere perfusion measurements. The root-mean-square error (RMSE) (mean prediction error between the measurement and the gold standard measurement), the root-mean-square deviation(RMSD) (mean prediction error between the measurement and the line of best fit in the regression analysis) and Lin’s concordance correlation coefficient [29] (concordance between the measurement and the gold standard measurement) were also determined. Student’s t-tests were used to assess mean differences of the FPA and MSM techniques with the reference microsphere perfusion measurements. Statistical software (SPSS, version 22, IBM, Armonk, NY) was used for all statistical analyses.

## Results

### Vitals and statistics

The FPA CT perfusion technique was validated in six swine (41.7 ± 10.2 kg) for a total of 44 perfusion measurements. Five acquisitions were excluded from the dataset since reference microsphere measurements were not available. Among the 39 successful perfusion measurements, 287 segments from different lobes of both left and right lungs (7 segments per animal on average) were used in the data analysis, as shown in **Table 2**.

**Table 2:** FPA and MSM mean pulmonary perfusion versus reference microsphere mean perfusion.

		Microsphere	FPA		MSM	
	Number of Samples	Reference Perfusion (ml/min/g)	CT Perfusion (ml/min/g)	P-value (α < 0.05)	CT Perfusion (ml/min/g)	P-value (α < 0.05)
	n	Mean ± SD	Mean ± SD		Mean ± SD	
**Right Lung**	**155**	**7.49 ± 3.51**	**7.40 ± 2.82**	**0.513**	**7.08 ± 2.78**	**0.128**
Right Cranial Lobe	12	6.79 ± 4.45	5.93 ± 3.36	0.038*	4.03 ± 1.91	0.005*
Accessory Lobe	39	7.88 ± 2.47	7.97 ± 4.67	0.776	8.23 ± 2.62	0.537
Right Middle Lobe	39	5.80 ± 2.60	6.44 ± 1.91	0.013*	7.32 ± 2.48	0.004*
Right Caudal Anterior Lobe	39	8.95 ± 2.79	8.49 ± 1.88	0.174	6.93 ± 1.80	<0.001*
Right Caudal Posterior Lobe	19	7.62 ± 5.24	7.04 ± 4.81	0.023*	6.64 ± 3.81	0.036*
**Left Lung**		**132**	**5.71 ± 4.09**	**5.63 ± 3.78**	**0.542**	**5.22 ± 3.16**	0.037*
Left Cranial Lobe	12	4.45 ± 2.21	4.22 ± 2.54	0.583	4.28 ± 2.58	0.728
Left Middle Lobe	27	7.76 ± 1.97	7.25 ± 2.27	0.126	7.97 ± 2.67	0.787
Left Caudal Superior Lobe	26	9.72 ± 4.26	9.14 ± 3.45	0.156	6.71 ± 2.61	<0.001*
Left Caudal Anterior Lobe	39	4.55 ± 2.95	4.65 ± 2.98	0.701	4.63 ± 2.03	0.814
Left Caudal Posterior Lobe	28	2.17 ± 3.37	2.76 ± 3.57	0.001*	2.28 ± 2.44	0.055
**Both Lungs**		**287**	**6.68 ± 3.89**	**6.59 ± 3.41**	**0.440**	**6.21 ± 3.08**	0.008*

Note.—SD: Standard Deviation.

1. The mean perfusion values are the average perfusion values of each lobe sample, the SD values are the perfusion value variations from the same sample, not the measurement errors.

2. The P-values are calculated by the paired-samples t-test statistics. The larger P-values indicate higher similarity between the two groups of data.

* Denotes mean CT perfusion values that are significantly different from reference microsphere perfusion values.

The overall heart rate and mean arterial pressure during all the CT perfusion acquisitions were 94.7 ± 18.9 bpm and 68.8 ± 22.8 mmHg, respectively. The intra-cluster correlation was calculated to be r = 0.19, indicating negligible correlation within intra-cluster measurements and statistical independence [30]. The effective sample size was found to be 28, showing that 28 independent individual subjects were equivalently used in this study [31].

### Lung mass validation

The average CT-measured lung mass and the actual ex-vivo mass were 455.4g (372.5 – 496.0g) and 434.3g (347.2 – 487.4g), respectively (p = 0.20, RMSE = 38.2g).

### Qualitative validation

**Fig 3:**
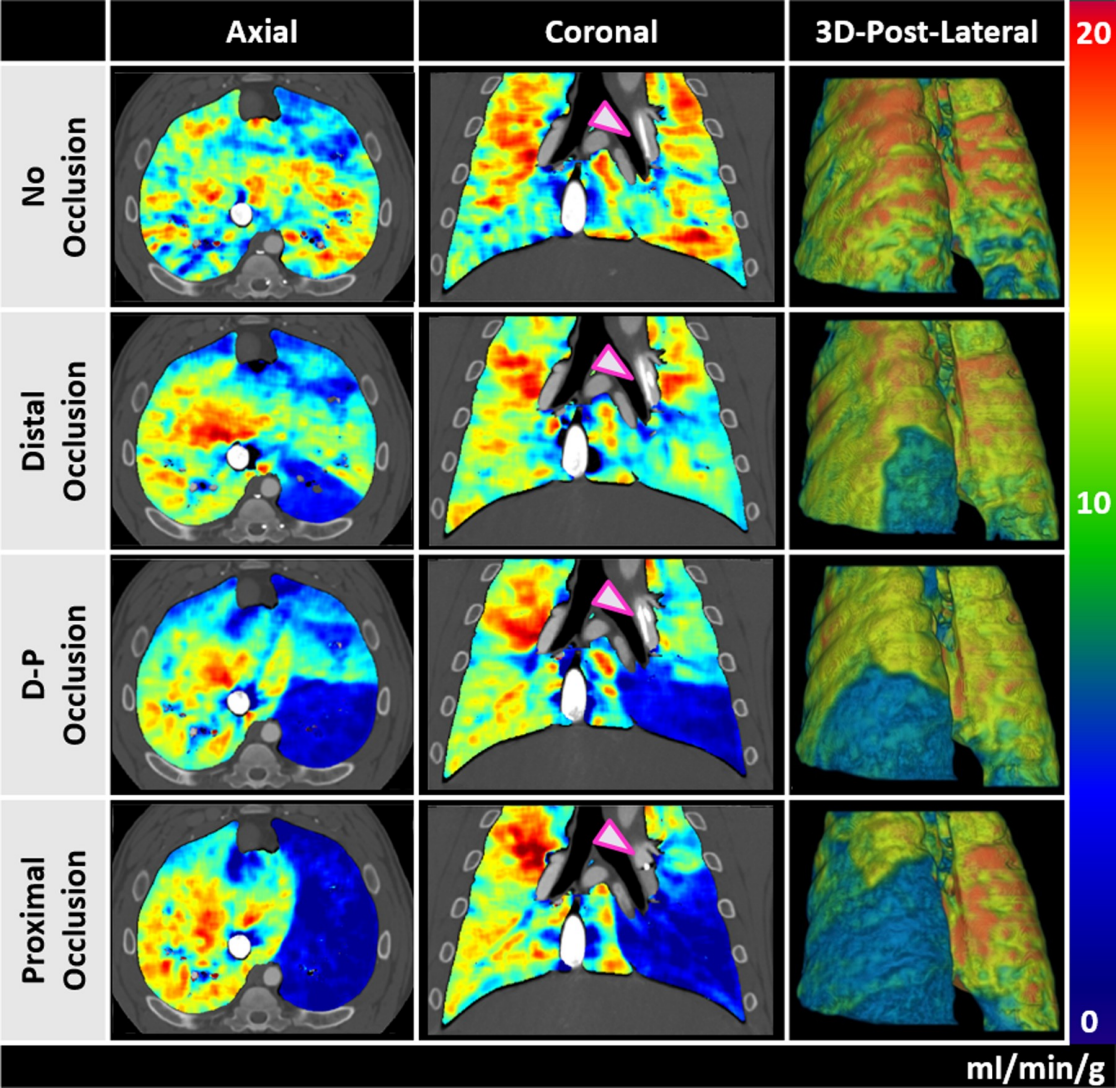
FPA pulmonary perfusion maps for different balloon occlusions. Axial, coronal, and 3D posterior-lateral views in the presence of no occlusion (first row), a distal occlusion (second row), a distal-to-proximal occlusion (third row) and a proximal occlusion (fourth row) are shown. The arrows indicate the location of the balloon for each occlusion. The color bar indicates perfusion in the range of 0–20 ml/min/g. The images were acquired at 400 mA.

**Fig 4:**
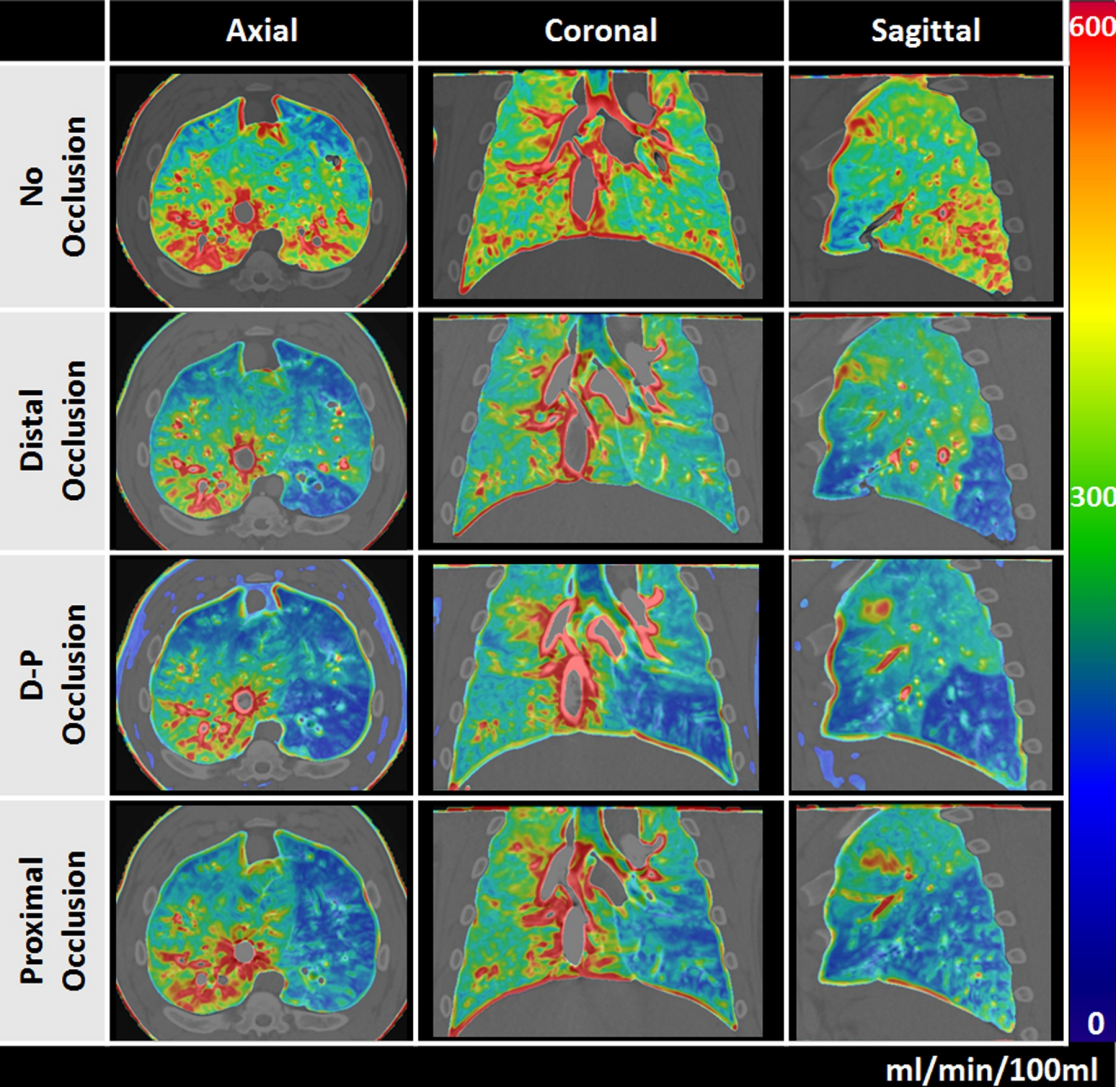
MSM pulmonary perfusion maps for different balloon occlusions. Axial, coronal, and sagittal views in the presence of no occlusion (first row), a distal occlusion (second row), a distal-to-proximal occlusion (third row) and a proximal occlusion (fourth row) are shown. The color bar indicates perfusion in the range of 0–600 ml/min/100ml. The images were acquired at 400 mA.

No significant image quality differences were found between images acquired using 400 and 200mA. Representative FPA and MSM pulmonary perfusion maps under 4 different balloon occlusion conditions acquired at 400 mA are shown in **Figs 3** and **4.** Additional FPA pulmonary perfusion maps with and without balloon occlusion acquired at 200 mA are shown in **S1 Fig**.

### Quantitative analysis

For all segments combined, the mean lung perfusion of the FPA and MSM techniques were 6.59 ± 3.41 (p = 0.44) and 6.21 ± 3.08 (p = 0.008) ml/min/g, respectively, while the mean microsphere perfusion was 6.68 ± 3.89 ml/min/g. Detailed pulmonary perfusion comparisons (n = 287) are summarized in **Tables 2** and **3**. Since the balloon occluded the arterial branch in the left caudal lobe, a reduction in perfusion can be found in the occluded regions in the left caudal anterior lobe (n = 39, FPA: 4.65 ± 2.98 vs. microsphere: 4.55 ± 2.95 in ml/min/g) and left caudal posterior lobe (n = 28, FPA: 2.76 ± 3.57 vs. microsphere: 2.17 ± 3.37 in ml/min/g) as compared to the other lobes. Except for the occluded regions, the mean perfusion values gradually decreased from the caudal lobe to the cranial lobe, indicating the change in the lung perfusion by gravitational effects, as shown in **Table 2**. Significant differences from the middle lobes, cranial lobes are mainly caused by the beam hardening artifacts by the contrast in vena cava and heart.

**Table 3:** FPA and MSM pulmonary perfusion regression versus reference microsphere perfusion.

Technique	Slope	Intercept	Pearson r	CCC	RMSE (ml/min/g)	RMSD (ml/min/g)
**FPA**						
**Right Lung (n = 155)**	0.69* (0.63, 0.76)	2.22* (1.68, 2.77)	0.85* (0.80, 0.89)	0.83* (0.77, 0.88)	1.81	0.87
**Left Lung (n = 132)**	0.85* (0.79, 0.91)	0.78* (0.34, 1.22)	0.92* (0.89, 0.94)	0.92* (0.88, 0.94)	1.61	0.58
**Both Lungs (n = 287)**	0.79* (0.75, 0.84)	1.32* (0.98, 1.67)	0.90* (0.87, 0.92)	0.89* (0.86, 0.91)	1.72	1.50
**MSM**						
**Right Lung (n = 155)**	0.36 (0.24, 0.47)	4.42 (3.49, 5.36)	0.45 (0.30, 0.57)	0.43 (0.29, 0.56)	3.38	2.48
**Left Lung (n = 132)**	0.59 (0.51, 0.68)	1.83 (1.24, 2.44)	0.77 (0.69, 0.84)	0.74 (0.65, 0.81)	2.69	2.05
**Both Lungs (n = 287)**	0.51 (0.44, 0.58)	2.78 (2.24, 3.33)	0.64 (0.57, 0.71)	0.62 (0.54, 0.69)	3.09	2.38

Note.—Data in parentheses are 95% confidence intervals. CCC: concordance correlation coefficient, RMSD: root-mean-square deviation, RMSE: root-mean-square error.

* Denotes FPA perfusion regression parameters that are significantly different than MSM perfusion regression parameters, as indicated by non-overlap of each 95% confidence interval, respectively.

**Fig 5:**
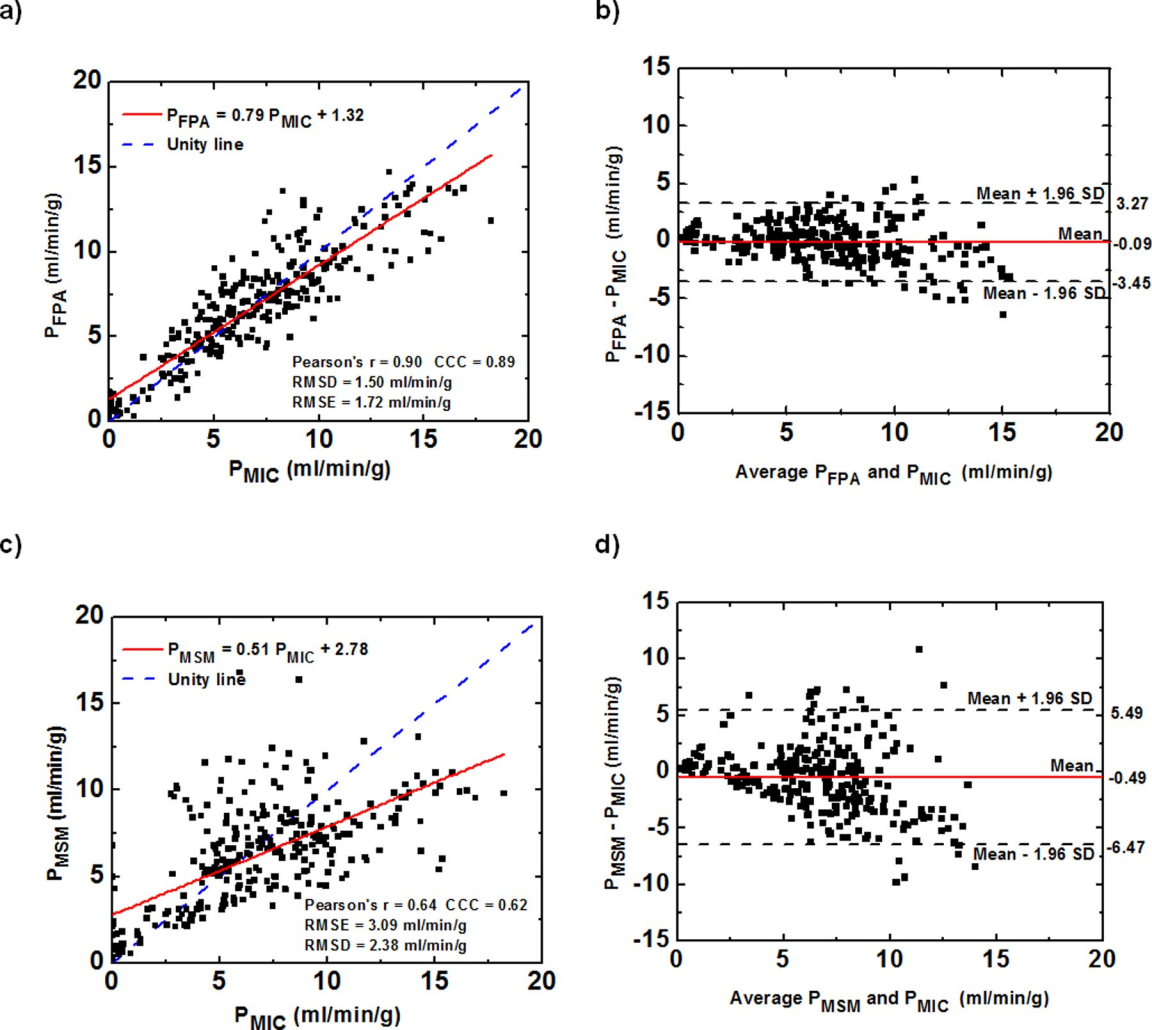
Quantitative analysis of FPA and MSM perfusion measurements. (a,c) Regression analysis comparing the result of the FPA and MSM perfusion measurements to the corresponding reference fluorescent microsphere perfusion measurements. (b,d) Bland-Altman analysis was performed for both techniques, with the limits of agreement. *P*_*FPA*_: FPA perfusion measurements, *P*_*MIC*_: Reference fluorescent microsphere measurements, *P*_*MSM*_: MSM perfusion measurements. RMSD = root-mean-square deviation, RMSE = root-mean-square error, CCC = concordance correlation coefficient.

FPA perfusion measurements (P_FPA_) were related to the reference fluorescent microsphere measurements (P_MIC_) by P_FPA_ = 0.79P_MIC_+1.32 (r = 0.90), with a concordance correlation coefficient (CCC) of 0.89, a root-mean-square deviation (RMSD) of 1.50 ml/min/g and a root-mean-square error (RMSE) of 1.72 ml/min/g (**Fig 5A**). MSM perfusion measurements (P_MSM_) were related to reference fluorescent microsphere perfusion measurements by P_MSM_ = 0.51P_MIC_+2.78 (r = 0.64), with a CCC of 0.62, RMSD of 2.38 ml/min/g and RMSE of 3.09 ml/min/g (**Fig 5C**). Both FPA and MSM measurements had a downward trend in the Bland-Altman plot (**Fig 5B** and **5D**), but the MSM technique showed worse correspondence at higher perfusion measurements. With respect to the two different tube currents, the overall measurement error was slightly lower for high tube currents as compared with the low tube currents. Specifically, for 400mA data, the RMSE and RMSD for the FPA technique were 1.37 and 1.29 ml/min/g, for the MSM technique were 2.69 and 2.14 ml/min/g, respectively. For 200 mA data, the RMSE and RMSD for the FPA technique were 2.34 and 1.92 ml/min/g, for the MSM technique were 3.87 and 2.87 ml/min/g, respectively.

**Fig 6:**
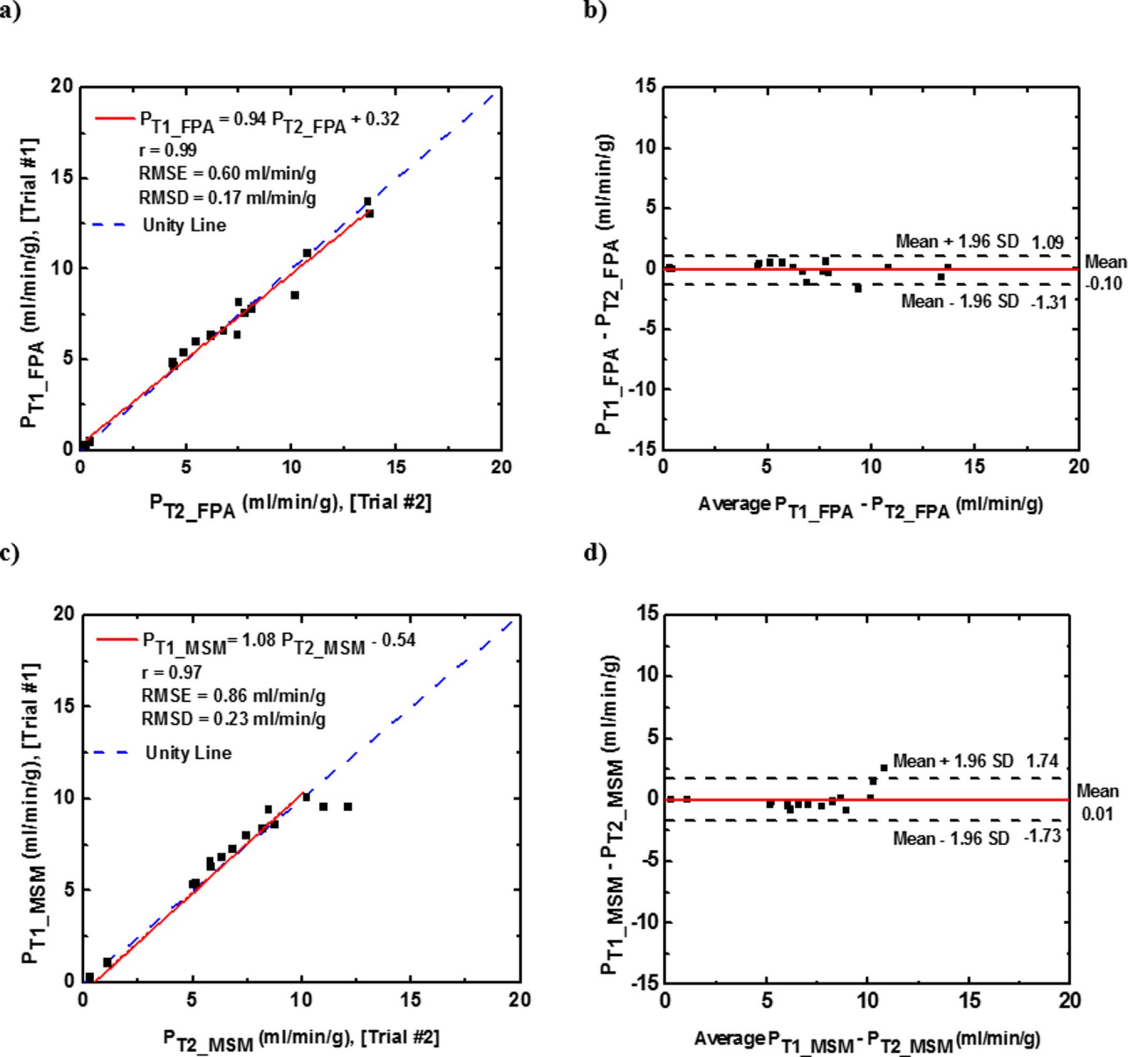
Repeatability analysis of FPA and MSM perfusion measurments. Two repeated contrast injections were made in two animals under the same perfusion condition. (a,c) Regression analysis comparing the result of the Trial #1 to Trial #2 using the FPA and MSM perfusion measurements. (b,d) Bland-Altman analysis was performed for both techniques, with the limits of agreement. *P*_*T1*_*FPA*_, *P*_*T2*_*FPA*_: FPA perfusion measurements, *P*_*T1*_*MSM*_, *P*_*T2*_*MSM*_: MSM perfusion measurements. RMSD = root-mean-square deviation, RMSE = root-mean-square error.

The repeatability analysis of the FPA and MSM perfusion measurements are shown in **Fig 6**. Two repeated contrast injections were made in two animals with the same occlusion. The interval between the two acquisitions, the average change in heart rate and blood pressure were 15 minutes, 2.18% and 12.75%, respectively. The results of the first (P_T1_) and the second (P_T1_) measurements were related by P_T1_FPA_ = 0.94 P_T2_FPA_ + 0.32 (r = 0.99, RMSE: 0.60 ml/min/g, RMSD: 0.17 ml/min/g) for the FPA technique and P_T1_MSM_ = 1.08 PT2_MSM− 0.54 (r = 0.97, RMSE: 0.86 ml/min/g, RMSD: 0.23 ml/min/g) for the MSM technique (**Fig6A** and **6C**). The repeatability for FPA and MSM measurements had negligible bias and lower variability (**Fig 6B** and **6D**) as compared to the accuracy measurements (**Fig 5**).

### Dose estimation

For the retrospectively acquired perfusion images at 200mA, the CT dose index for the MSM techniques was 138.7mGy. For the FPA technique, if the two volume scans were acquired prospectively, the CTDI would have been 8.4mGy. All other dose metrics are reported in **Table 1**.

## Discussion

The results indicate that the FPA technique can accurately measure pulmonary perfusion on a voxel-by-voxel basis as indicated by the good agreement with reference fluorescent microsphere perfusion measurement. The FPA technique also has high precision for repeated perfusion measurements. Furthermore, the pulmonary perfusion measurement can be made using only two volume scans, indicating significant potential for dose reduction with its prospective implementation. Moreover, the second volume acquisition for the pulmonary perfusion measurement could potentially be used for CT pulmonary angiography; hence, the FPA technique has the potential to provide anatomical and physiological assessment of lung disease following a single contrast injection, while also reducing the radiation dose, although further validation is still necessary.

Previously reported MSM techniques [12–14] use small regions-of-interest (ROIs) for perfusion measurements, resulting in underestimation of the perfusion due to the problem of contrast loss during the measurement time. Our results agree with previous reports showing the underestimation of the MSM technique, especially at higher perfusion rates. Deconvolution-based techniques have been used to resolve the underestimation issue by estimating the venous contrast outflow over the measurement time [32, 33]. However, these techniques require the entire contrast-pass curve and involve high radiation doses. On the other hand, for the proposed FPA technique, the expansion of the compartment size to encompass the whole lobe enables the possibility of measuring blood flow using only two volume scans and minimizes the problem of contrast loss over the measurement time, improving the accuracy of the perfusion measurement.

Apart from the measurement accuracy, the prospective implementation of the FPA technique with two-volume scan is the most critical issue to be solved in reducing the radiation dose. A prospective timing protocol for FPA technique has recently been reported for the optimal capture of the two-volume scans at the base and peak of the aortic enhancement [34]. It was shown that V1 can be acquired based on a bolus-tracking technique and V2 can be acquired after a pre-defined time-to-peak delay based on contrast injection time interval. A robust relation between the contrast injection time and the aortic enhancement time-to-peak delay was investigated to predict V2 timing, which can be further studied for the pulmonary system. A previous report has shown that accurate perfusion measurements can be made using the FPA technique for different timing protocols of the first (V1) and second (V2) volume scans [34]. Furthermore, a diluted test-bolus technique [35] was also reported to prospectively determine the timing for the two-volume scans, however, with slightly increased contrast and radiation dose. By injecting the same amount of diluted contrast as the actual contrast bolus, the contrast arrival timing (V1) and maximal enhancement timing (V2) can be monitored and used for the prospective FPA volume scans [35].

The breath-hold difficulty is another limitation in clinical practice due to the length of time required for image acquisition. Previously reported dynamic CT perfusion techniques [4, 7–9, 12, 13, 15, 33, 36] require sampling most of the contrast pass curve with a large number of volume acquisitions for approximately 30 seconds. Images are often acquired during shallow breathing, resulting in major motion artifacts. Fortunately, given the unique two-volume sampling protocol of the FPA technique, its prospective implementation will only require a short breath hold of approximately 10 seconds. The relatively short breath-hold time is expected to substantially reduce the motion misregistration artifacts and make the FPA technique more feasible for patients with lung disease.

The effective radiation dose of dynamic CT perfusion techniques for volume CT scanners with equivalent 16-cm detector has been reported to be in the range of 4.55-50mSv [7–9, 13, 15–17], using a continuous dynamic volume scanning mode. If implemented prospectively, the effective radiation dose for the two-volume FPA technique is expected to be approximately 3.98mSv at 200mA using the SSDE and a chest conversion factor of 0.014mGy∙cm [26]. Future studies are necessary to optimize the radiation dose associated with the FPA technique, including optimization of tube current, beam energy, and other scanning parameters. Nevertheless, significant potential exists for radiation dose reduction in the proposed dynamic FPA CT perfusion technique.

### Limitations

Despite improvements in quantitative pulmonary perfusion, our study had limitations. Firstly, the comparison between the physical tissue samples and the corresponding digital segmentations is challenging. Potential errors were minimized using anatomical landmarks for improved registration during the regional segmentation process. Larger physical sample sizes could help to reduce the measurement variation caused by spatial misregistration and motion. Secondly, images were acquired at a relatively high tube current, which is not necessary for accurate perfusion measurement. Therefore, additional radiation dose optimization studies will be required. Thirdly, highly attenuating contrast material in the vena cava, heart, and aorta generated significant streaking artifacts that impacted all adjacent lobes. These artifacts potentially degrade the CT perfusion measurement accuracy and precision. Further contrast dose reduction and the use of beam hardening correction algorithms [37, 38] can be helpful in reducing such artifacts. Fourthly, the z-axis range was limited to 16 cm, which is smaller than the range necessary for whole lung imaging in human subjects. However, using an individual lobe as the compartment would be sufficient for the FPA model. Finally, image quality degradation is expected in the periphery of the scan range. However, this effect was difficult to quantify since all the measurements were made at approximately the same distance from the iso-center.

## Conclusions

The proposed FPA technique can provide pulmonary perfusion measurement using two volume scans. In summary, the first-pass analysis technique for pulmonary perfusion measurement was validated in a swine model and has the potential to reduce the radiation dose associated with dynamic CT perfusion for assessment of pulmonary disease.

## Supporting information

S1 Fig FPA pulmonary perfusion maps for different balloon occlusions. Axial, coronal, and 3D posterior views in the presence of no occlusion (first row) and a distal occlusion (second row) are shown. The color bar indicates perfusion in the range of 0–20 ml/min/g. Images were acquired at 200 mA.
(TIF)

S1 File Appendix for first-pass analysis perfusion technique derivation. (DOCX)

S2 File Raw data spreadsheet for all perfusion measurements. (PDF)
