# Supplementary figures and images for: Dynamic pulmonary CT perfusion using first-pass analysis technique with only two volume scans: Validation in a swine model
Source: PLoS One. 2020 Feb 12;15(2):e0228110. doi: 10.1371/journal.pone.0228110 (PMC7015394; doi:10.1371/journal.pone.0228110)

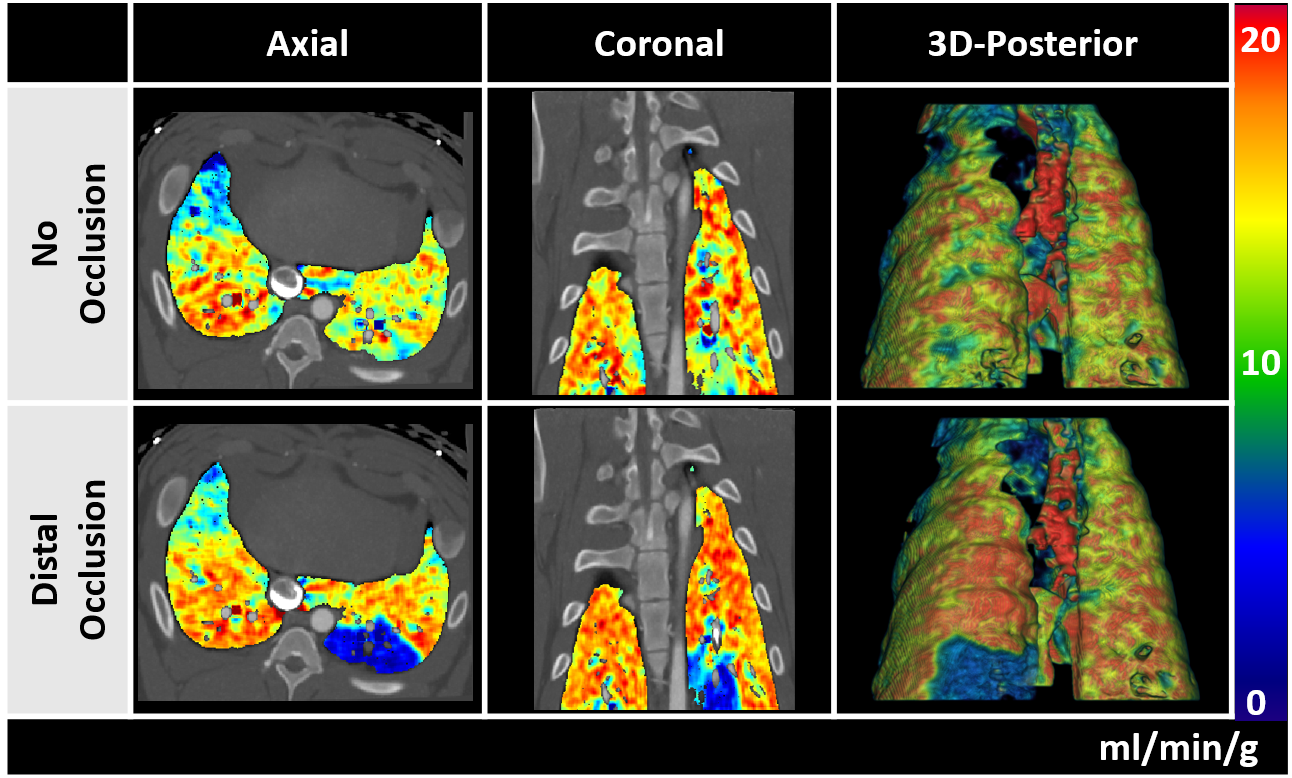

Supplement: S1 Fig — Axial, coronal, and 3D posterior views in the presence of no occlusion (first row) and a distal occlusion (second row) are shown. The color bar indicates perfusion in the range of 0–20 ml/min/g. Images were acquired at 200 mA. (TIF) [file pone.0228110.s001.tif]
