# Supplementary material for: Dynamic pulmonary CT perfusion using first-pass analysis technique with only two volume scans: Validation in a swine model
Source: PLoS One. 2020 Feb 12;15(2):e0228110. doi: 10.1371/journal.pone.0228110 (PMC7015394; doi:10.1371/journal.pone.0228110)
Supplement: S1 File — (DOCX) [file pone.0228110.s002.docx]

# Appendix for first-pass analysis perfusion technique derivation

Blood Flow Measurement by FPA Technique

The FPA technique measures the accumulation of iodine mass ($\boldsymbol{M}_{\boldsymbol{c}}\left( \boldsymbol{t} \right)\boldsymbol{, mg}$) within the perfusion compartment ([1](#_ENREF_1), [2](#_ENREF_2)), as represented by:

$\boldsymbol{M}_{\boldsymbol{c}}\left( \boldsymbol{t} \right)\boldsymbol{=}\int_{\boldsymbol{0}}^{\boldsymbol{t}} \boldsymbol{[Q}_{\boldsymbol{in}}\left( \boldsymbol{t} \right)\boldsymbol{C}_{\boldsymbol{in}}\left( \boldsymbol{t} \right)\boldsymbol{-}\boldsymbol{Q}_{\boldsymbol{out}}\left( \boldsymbol{t} \right)\boldsymbol{C}_{\boldsymbol{out}}\boldsymbol{(t)]dt}$ ***Eq. (A.1)***

where $\boldsymbol{M}_{\boldsymbol{c}}\left( \boldsymbol{t} \right)$ is the accumulated iodine mass (***mg***) within the compartment; $\boldsymbol{Q}_{\boldsymbol{in}}\left( \boldsymbol{t} \right)\boldsymbol{,}\boldsymbol{Q}_{\boldsymbol{out}}\boldsymbol{(t)}$are the inflow and outflow for the compartment ($\boldsymbol{ml/min)}$; $\boldsymbol{C}_{\boldsymbol{in}}\left( \boldsymbol{t} \right)\boldsymbol{,}\boldsymbol{C}_{\boldsymbol{out}}\boldsymbol{(t)}$ are the incoming and outgoing concentration of iodine ($\boldsymbol{mg/ml)}$.

For pulmonary flow measurement, the accumulation of contrast mass is merely caused by pulmonary circulation. Assuming that the measurement is made before the contrast outflow, ***A. 1*** can be simplified as:

$\boldsymbol{M}_{\boldsymbol{c}}\left( \boldsymbol{t} \right)\boldsymbol{=}\int\boldsymbol{Q}_{\boldsymbol{P}}\left( \boldsymbol{t} \right)\boldsymbol{C}_{\boldsymbol{pa}}\left( \boldsymbol{t} \right)\boldsymbol{dt}$ ***Eq. (A.2)***

where $\boldsymbol{Q}_{\boldsymbol{P}}\left( \boldsymbol{t} \right)$ is the pulmonary blood flow, $\boldsymbol{C}_{\boldsymbol{pa}}\left( \boldsymbol{t} \right)$ is the iodine concentration ($\boldsymbol{mg/ml)}$within the pulmonary artery.

Taking derivative on both sides of ***A.2***, the pulmonary flow ($\boldsymbol{Q}_{\boldsymbol{P\_ave}}\boldsymbol{,ml/min}$) is given by:

$\boldsymbol{Q}_{\boldsymbol{P\_ave}}\boldsymbol{=}\frac{\boldsymbol{1}}{\boldsymbol{C}_{\boldsymbol{pa\_ave}}} \frac{\boldsymbol{\Delta M}_{\boldsymbol{c}}}{\boldsymbol{\Delta t}}$ ***Eq. (A.3)***

where $\boldsymbol{\Delta}\boldsymbol{M}_{\boldsymbol{c}}$ is the overall contrast mass change within the compartment over the measurement period ($\boldsymbol{\Delta t}$), $\boldsymbol{C}_{\boldsymbol{pa\_ave}}$ is the average input concentration of pulmonary artery.

Voxel-by-voxel Blood Flow Normalization

Since the contrast concentration change ($\boldsymbol{\Delta HU}$) within the entire compartment is approximately proportional to the contrast material mass change, i.e. the average pulmonary flow ($\boldsymbol{Q}_{\boldsymbol{P\_ave}}$), the voxel-by-voxel contrast concentration change ($\boldsymbol{\Delta H}\boldsymbol{U}_{\boldsymbol{x,y,z}}$) can be used to estimate pulmonary blood flow on a voxel-by-voxel basis (${\boldsymbol{Q}_{\boldsymbol{P}}}_{\boldsymbol{x,y,z}}\boldsymbol{,ml/min}$) as:

${\boldsymbol{Q}_{\boldsymbol{P}}}_{\boldsymbol{x,y,z}}\boldsymbol{=}\boldsymbol{Q}_{\boldsymbol{P\_ave}}\frac{\boldsymbol{\Delta H}\boldsymbol{U}_{\boldsymbol{x,y,z}}}{\boldsymbol{\Delta HU}}$ ***Eq. (A.4)***

Voxel-by-voxel Perfusion Normalization

The pulmonary blood flow is normalized to tissue mass to account for its spatial heterogeneity. The fraction of non-air tissue ($\boldsymbol{T}_{\boldsymbol{f}}$**, %**) is calculated from the non-contrast enhanced image V1 with decomposition of the pure soft tissue (greater than 50 HU) and air $($less than -1000 HU) ([3](#_ENREF_3), [4](#_ENREF_4)). The mass of each voxel ($\boldsymbol{M}_{\boldsymbol{x, y,z}}$) is the product of tissue fraction $\boldsymbol{(T}_{\boldsymbol{f}})$, voxel size ($\boldsymbol{Voxel}_{\boldsymbol{x,y,z}}\boldsymbol{,}\boldsymbol{cm}^{\boldsymbol{3}}$) and the non-air tissue density (**1.04** $\boldsymbol{g/}\boldsymbol{cm}^{\boldsymbol{3}}$) ([4](#_ENREF_4)) as:

$\boldsymbol{T}_{\boldsymbol{f}}\boldsymbol{=}\frac{\boldsymbol{HU}_{\boldsymbol{x,y,z}}\boldsymbol{-}\boldsymbol{HU}_{\boldsymbol{Air}}}{\boldsymbol{HU}_{\boldsymbol{Tissue}}\boldsymbol{-}\boldsymbol{HU}_{\boldsymbol{Air}}}$ ***Eq. (A.5)***

$\boldsymbol{M}_{\boldsymbol{x, y,z}}\boldsymbol{=}\boldsymbol{T}_{\boldsymbol{f}}\boldsymbol{\times}\boldsymbol{Voxel}_{\boldsymbol{x,y,z}}\boldsymbol{\times}$ ***1.04Eq. (A.6)***

where $\boldsymbol{HU}_{\boldsymbol{x,y,z}}$ represents the Hounsfield unit number per each voxel in V1 image, $\mathrm{HU}_{\mathrm{Tissue}}$ = 50 HU, $\mathrm{HU}_{\mathrm{Air}}=-1000HU.$ $\boldsymbol{M}_{\boldsymbol{x, y,z}}$ is the tissue mass per voxel in gram, $\boldsymbol{T}_{\boldsymbol{f}}\boldsymbol{\geq}1$is set as 1.

The ultimate voxel-by-voxel pulmonary perfusion (${\boldsymbol{P}_{\boldsymbol{P}}}_{\boldsymbol{x,y,z}}\boldsymbol{,ml/min/g}$) s derived as:

${\boldsymbol{P}_{\boldsymbol{P}}}_{\boldsymbol{x,y,z}}\boldsymbol{=}\frac{{\boldsymbol{Q}_{\boldsymbol{P}}}_{\boldsymbol{x,y,z}}}{\boldsymbol{M}_{\boldsymbol{x, y,z}}}$ ***Eq. (A.7)***

1. Molloi S, Zhou Y, Kassab GS. Regional volumetric coronary blood flow measurement by digital angiography: in vivo validation. Acad Radiol. 2004;11(7):757-66.

2. Molloi S, Bednarz G, Tang J, Zhou Y, Mathur T. Absolute volumetric coronary blood flow measurement with digital subtraction angiography. Int J Cardiovasc Imaging. 1998;14(3):137-45.

3. Chon D, Beck KC, Larsen RL, Shikata H, Hoffman EA. Regional pulmonary blood flow in dogs by 4D-X-ray CT. J Appl Physiol (1985). 2006;101(5):1451-65.

4. Busse N, Erwin W, Pan T. Evaluation of a semiautomated lung mass calculation technique for internal dosimetry applications. Med Phys. 2013;40(12):122503.
