# Supplementary material for: Dynamic pulmonary CT perfusion using first-pass analysis technique with only two volume scans: Validation in a swine model
Source: PLoS One. 2020 Feb 12;15(2):e0228110. doi: 10.1371/journal.pone.0228110 (PMC7015394; doi:10.1371/journal.pone.0228110)
Supplement: S2 File — (PDF) [file pone.0228110.s003.pdf]

Animal ID			Acquisition #	Microspehre	FPA	MSM
	All data was used for Tables 1, 2, 3 and Figure 5				ml/min/g	
#1	Used for Fig 1	Right Caudal Base Posterior	1	0.539	0.4617	1.1064
(400mA)	Used for Fig 6		2	0.508	0.4381	1.0687
	Used for Fig 6		3	0.482	0.4289	1.0876
			4			
			5	0.45	0.3961	0.8953
			6	0.492	0.4683	0.9054
			7	0.414	0.424	0.9096
			8	0.382	0.3276	0.6526
		Accessory Lobe	1	7.066	6.5881	8.722
	Used for Fig 6		2	7.204	6.7943	8.5849
	Used for Fig 6		3	5.942	6.425	8.7501
			4			
			5	7.057	7.3869	8.5401
			6	8.129	8.1862	8.8776
			7	6.58	8.429	8.6196
			8	8.392	6.4849	8.1496
		Right Middle Lobe	1	2.994	5.3771	10.221
			2	3.025	4.888	10.0599
			3	2.753	4.5311	9.768
			4			
			5	3.433	2.9676	9.4153
			6	3.619	5.8257	8.9308
			7	2.896	2.8024	9.6018
			8	3.744	4.7448	8.4239
		Right Caudal Anterior	1	6.274	6.3138	6.3507
	Used for Fig 6		2	6.338	6.1767	6.788
	Used for Fig 6		3	5.742	5.4058	7.192
			4			
			5	6.476	6.3541	6.7763
			6	7.256	7.509	6.6356
			7	5.784	6.5426	7.4296
			8	7.476	6.1674	6.7145
		Right Caudal Superior_Posterior	1	8.972	7.5517	7.4504
	Used for Fig 6		2	9.241	7.8132	7.9778
	Used for Fig 6		3	7.897	7.3815	8.3949
			4			
			5	8.612	7.7029	7.4358
			6	9.844	8.1644	7.1927
			7	8.1	7.762	8.0976
			8	10.546	7.2117	7.3056
		Left Caudal Base Posterior	1	0.005	0.2787	0.2716
	Used for Fig 6		2	0.001	0.2367	0.2769
	Used for Fig 6		3	0.001	0.1019	0.331
			4			
			5	0.002	0.0718	0.2389
			6	0.001	0.2045	0.2421
			7	0.002	0.2134	0.261
			8	0.004	0.1288	0.2269
		Left Middle Lobe	1	4.868	4.8205	5.8193
	Used for Fig 6		2	4.884	4.3895	6.2956
	Used for Fig 6		3	4.807	4.2655	6.696
			4			
			5	5.365	4.1408	5.8127
			6	5.875	5.1476	5.9032
			7	4.835	3.9783	6.5849
			8	6.27	4.5742	
		Left Caudal Anterior	1	4.941	4.6594	5.0006
	Used for Fig 6		2	4.382	4.4584	5.3532
	Used for Fig 6		3	4.638	4.2239	5.3639
			4			
			5	4.785	4.8586	5.5224
			6	5.407	5.1512	5.2625
			7	4.203	5.0855	5.565
			8	5.355	4.306	4.7945
		Left Caudal Superior_Posterior	1	4.608	5.9763	5.1431
	Used for Fig 6		2	4.986	5.4734	5.3647
	Used for Fig 6		3	5.318	5.5924	5.7378
			4			
			5	5.452	5.9941	5.5627
			6	6.4	6.3016	5.2831
			7	5.306	6.0473	8.0976
			8	6.863	5.4081	5.3246
#2		Accessory Lobe	1	8.957	8.6593	6.6694
(400mA)			2	9.504	10.5437	7.2131
			3	10.29	10.1706	7.9084
			4	11.268	9.4528	8.1475
			5	8.04	8.5927	8.1186
			6	8.649	9.1984	6.8817

			7	9.648	8.5061	7.0472
			8	8.403	7.8234	7.086
		**Right Middle Lobe**	1	7.009	6.6303	9.0901
			2	7.816	7.1178	7.6496
			3	8.545	7.4215	8.6863
			4	8.809	9.3995	7.9444
			5	6.764	4.9487	8.318
			6	6.94	6.7799	7.1673
			7	7.386	7.5031	7.0793
			8	6.799	7.9546	7.0112
		**Right Caudal Anterior**	1	9.326	8.5809	6.8255
			2	10.77	10.0522	7.4327
			3	11.507	9.6103	7.942
			4	13.215	10.0845	8.4594
			5	9.288	9.0405	8.4273
			6	9.943	9.5588	7.2687
			7	11.05	9.0369	7.4489
			8	9.657	8.6105	7.3923
		**Left Caudal Base Posterior**	1	5.164	5.4068	5.4384
			2	0.325	0.6294	0.497
			3	0.482	1.1962	0.6078
			4	0.427	0.5685	0.6038
			5	6.282	6.3044	7.8655
			6	2.812	3.8586	5.1438
			7	0.862	0.664	0.5467
			8	1.874	1.3276	3.1471
		**Left Middle Lobe**	1	7.939	7.3178	6.0585
			2	8.445	6.8649	6.1638
			3	9.951	7.1456	6.8307
			4	8.712	7.6096	6.8707
			5	7.129	6.9029	7.3469
			6	7.814	7.1337	6.4415
			7	8.701	6.7361	6.386
			8	7.621	6.4624	6.3911
		**Left Caudal Anterior**	1	6.7	6.2266	5.4852
			2	1.165	1.405	1.835
			3	0.233	1.5918	1.6562
			4	0.473	1.0058	1.7967
			5	6.898	6.281	6.6246
			6	4.794	5.9738	5.1785
			7	4.197	4.6059	4.6158
			8	4.293	4.8316	5.0867
**#3**	**Used for Figs 3, 4**	**Left Caudal Lobe Anterior**	**1**	4.421	6.4452	7.5963
**(400mA)**	**Used for Figs 3, 4**		**2**	5.955	5.5926	4.4841
	**Used for Figs 3, 4**		**3**	8.641	4.9502	3.8747
	**Used for Figs 3, 4**		**4**	0.096	1.0095	4.2967
			5			
	**Used for Figs 3, 4**	**Left Cranial Lobe Anterior**	**1**	6.517	7.07048	10.4513
	**Used for Figs 3, 4**		**2**	6.994	9.8805	6.8274
	**Used for Figs 3, 4**		**3**	9.478	7.192	6.437
	**Used for Figs 3, 4**		**4**	5.166	3.9696	5.9569
			5			
	**Used for Figs 3, 4**	**Left Caudal Lobe Superior Posterior**	**1**	14.351	13.738	8.1699
	**Used for Figs 3, 4**		**2**	11.775	11.7044	6.1163
	**Used for Figs 3, 4**		**3**	15.224	10.0415	5.3823
	**Used for Figs 3, 4**		**4**	10.155	7.8173	6.2469
			5			
	**Used for Figs 3, 4**	**Right Cranial Lobe**	**1**	13.193	12.0175	8.0948
	**Used for Figs 3, 4**		**2**	11.146	10.3126	5.9292
	**Used for Figs 3, 4**		**3**	15.356	11.0592	6.0029
	**Used for Figs 3, 4**		**4**	10.232	9.3028	5.7527
			5			
	**Used for Figs 3, 4**	**Accessory Lobe**	**1**	7.443	11.781	12.4284
	**Used for Figs 3, 4**		**2**	7.814	8.2565	10.1311
	**Used for Figs 3, 4**		**3**	10.525	9.3487	9.2846
	**Used for Figs 3, 4**		**4**	5.97	7.7819	8.6696
			5			
	**Used for Figs 3, 4**	**Right Caudal Lobe Anterior**	**1**	9.288	13.0577	11.1512
	**Used for Figs 3, 4**		**2**	9.289	12.7008	8.1593
	**Used for Figs 3, 4**		**3**	12.466	10.245	6.4949
	**Used for Figs 3, 4**		**4**	6.937	8.412	7.7288
			5			
	**Used for Figs 3, 4**	**Right Middle Lobe**	**1**	5.433	8.5208	11.7918
	**Used for Figs 3, 4**		**2**	5.734	6.7293	7.7951
	**Used for Figs 3, 4**		**3**	7.746	7.4703	6.559
	**Used for Figs 3, 4**		**4**	4.145	6.8196	8.5004
			5			
**#4**		**Left Caudal Lobe Anterior**	1	1.198	0.3089	1.6081
**(400mA)**			2	5.215	5.751	3.6129
			3	6.097	5.8566	3.6372

			4	3.133	2.5145	2.541
			5	4.041	4.4574	3.3633
			6	1.324	1.1803	1.3335
			7	1.314	1.2414	1.1231
			8	3.716	1.8604	2.411
		**Left Caudal Base Lateral**	1	9.098	10.6589	5.3464
			2	0.018	1.443	0.8393
			3	0.159	0.6888	1.003
			4	1.62	1.7929	2.5724
			5	5.89	7.4189	4.315
			6	5.693	6.387	3.6279
			7	5.108	4.0834	3.0668
			8	1.66	3.7796	3.1154
		**Left Cranial Lobe Anterior**	1	2.013	2.0126	2.1536
			2	2.954	2.2712	2.1069
			3	3.646	2.7115	2.4588
			4	3.965	3.553	3.0743
			5	2.603	2.6509	2.9401
			6	2.61	3.6367	3.0042
			7	3.27	1.4088	2.7986
			8	4.153	4.3208	3.1394
		**Left Caudal Lobe Superior Posterior**	1	9.31	11.298	5.6433
			2	5.887	9.5885	4.9853
			3	6.426	8.8235	4.6816
			4	3.411	2.5291	2.2023
			5	8.983	11.0348	5.9524
			6	8.66	9.1317	4.9814
			7	8.081	6.2109	4.2233
			8	7.487	4.3322	3.2902
		**Right Cranial Lobe**	1	2.556	2.8247	2.1948
			2	3.502	2.4465	2.118
			3	4.225	2.835	2.5797
			4	5.155	4.4771	3.2893
			5	3.111	3.816	3.0885
			6	3.475	3.6528	3.0917
			7	3.764	3.4988	2.8081
			8	5.721	4.9384	3.3927
		**Accessory Lobe**	1	5.03	5.5259	5.0213
			2	6.613	7.0737	4.7836
			3	7.616	7.1646	4.9655
			4	9.159	7.2221	4.5741
			5	5.863	6.2638	4.3518
			6	6.363	7.0144	4.2774
			7	6.58	4.9652	3.9515
			8	7.636	5.9568	4.4554
		**Right Caudal Lobe Anterior**	1	5.864	7.6018	4.2957
			2	8.057	7.5236	4.2734
			3	9.706	8.1476	4.4931
			4	10.721	9.6063	4.8914
			5	7.22	8.3471	4.7125
			6	7.562	8.7971	4.4918
			7	8.097	7.0298	4.2395
			8	9.948	8.2162	4.4523
		**Right Middle Lobe**	1	2.509	3.7335	2.8122
			2	3.703	3.9561	2.7775
			3	4.463	4.7991	3.2032
			4	5.249	5.518	3.8947
			5	3.339	4.9487	3.9089
			6	3.336	5.2053	3.8299
			7	4.147	4.6085	3.4832
			8	5.664	5.3379	3.7682
**#5**		**R Middle Lobe**	1			
**(200mA)**			2	6.043	8.002	11.609
	**Used for S1 Fig**		**3**	4.992	6.9133	6.8782
	**Used for S1 Fig**		**4**	4.823	6.7724	6.6435
			5	4.244	5.972	6.4271
	**Used for Fig6**		**6**	5.424	7.452	6.5872
	**Used for Fig6**		**7**	5.672	6.3413	5.8105
			8	4.75	7.3426	6.8125
		**L Middle Lobe**	1			
			2	8.71	9.175	16.3622
	**Used for S1 Fig**		**3**	7.524	7.3738	10.5244
	**Used for S1 Fig**		**4**	8.077	10.3211	11.2795
			5	7.569	8.4928	10.4003
	**Used for Fig6**		**6**	9.231	10.1977	11.1532
	**Used for Fig6**		**7**	9.443	8.5511	9.5724
			8	8.25	10.0834	12.1165
		**R Lower Lobe Anterior**	1			
			2	7.176	7.6524	11.4099
	**Used for S1 Fig**		**3**	6.433	8.4987	6.2307

	Used for S1 Fig		4	6.344	8.1488	7.2111
			5	5.661	7.6151	6.6328
	Used for Fig6		6	7.198	7.5168	7.2672
	Used for Fig6		7	7.376	8.1252	6.831
			8	6.186	4.9025	9.8735
		L Lower Lobe Anterior	1			
			2	14.253	12.2405	13.0772
	Used for S1 Fig		3	12.998	11.4513	8.3761
	Used for S1 Fig		4	14.501	13.9679	10.364
			5	13.838	12.7037	9.8183
	Used for Fig6		6	16.525	13.4797	9.9781
	Used for Fig6		7	16.913	13.741	9.5507
			8	15.072	13.0359	10.9955
		R Accessory Lobe	1			
			2	5.96	7.799	16.8044
	Used for S1 Fig		3	5.089	5.8834	10.5494
	Used for S1 Fig		4	5.027	9.0316	9.9803
			5	4.438	7.5666	9.1172
	Used for Fig6		6	5.49	8.1088	9.3608
	Used for Fig6		7	5.346	7.7463	8.4882
			8	4.332	6.1368	11.5842
		R Lower Lobe Base	1			
			2	11.719	11.9372	12.7957
	Used for S1 Fig		3	12.176	11.5094	8.489
	Used for S1 Fig		4	13.649	13.1659	10.3041
			5	13.15	12.7261	9.8143
	Used for Fig6		6	15.278	13.655	10.2079
	Used for Fig6		7	16.194	13.7399	9.8967
			8	14.1974	13.403	10.8062
		L Lower Lobe Base Anterior	1			
			2	0.036	1.4731	4.9904
	Used for S1 Fig		3	8.298	13.5963	8.6063
	Used for S1 Fig		4	0.089	1.5363	2.2629
			5	5.945	6.6542	6.209
	Used for Fig6		6	7.859	10.7864	8.3458
	Used for Fig6		7	8.574	10.8804	8.2164
			8	0.018	0.1036	6.777
#6		R Middle Lobe	1	9.052	11.0782	11.5727
(200mA)			2			
			3	15.047	11.121	9.8372
			4	10.35	9.0317	7.7409
			5	9.384	7.676	7.3905
			6	8.54	6.8813	6.646
			7			
		L Middle Lobe	1	7.637	12.3228	9.0212
			2			
			3	13.652	10.0187	9.5314
			4	10.281	8.9826	6.526
			5	8.497	8.21	6.1237
			6	7.547	4.5842	4.9944
			7			
		R Lower Lobe Anterior	1	12.05	12.4957	9.1972
			2			
			3	18.234	11.8465	9.8061
			4	14.397	9.5187	6.4646
			5	11.849	8.9412	5.9078
			6	10.924	7.242	5.1069
			7			
		L Lower Lobe Anterior	1	4.747	5.1656	5.3888
			2			
			3	12.497	8.451	7.8507
			4	8.978	6.7968	5.3023
			5	7.354	6.133	4.2751
			6	5.264	3.8193	3.4011
			7			
		R Accessory Lobe	1	9.935	12.0258	11.9787
			2			
			3	15.853	10.7221	10.9945
			4	13.47	9.2074	9.5624
			5	10.952	9.1582	8.5373
			6	9.757	6.0686	7.8982
			7			
		R Lower Lobe Base	1	9.123	11.1076	10.5431
			2			
			3	9.998	8.3319	8.926
			4	7.119	4.7561	7.5084
			5	5.13	6.6931	7.0586
			6	3.819	5.3536	5.8663
			7			
		L Lower Lobe Base Anterior	1	13.358	14.7099	8.9409
			2			
			3	0.008	1.7588	2.0996
			4	0.009	0.9759	0.8415
			5	0.037	1.6578	1.5999
			6	0.01	0.7393	0.744
			7			
		**Total Number**	**324**	**287**	**287**	
		**Mean Both Lungs**		**6.6768**	**6.5859**	**6.2088**
		**STD Both Lungs**		**3.8869**	**3.4093**	**3.0841**
